# Patient and Family Perspectives on Statutory Duty of Candour Following Serious Adverse Patient Safety Events

**DOI:** 10.1111/hex.70800

**Published:** 2026-08-31

**Authors:** Corey Adams, Lorelle Bowditch, Mia Bierbaum, Ramya Walsan, Jennifer Morris, Elizabeth Manias, Nicole Youngs, Lanii Birks, Jeffrey Braithwaite, Ashfaq Chauhan, Peter Hibbert, Ramesh Walpola, Reema Harrison

**Affiliations:** ^1^ Australian Institute of Health Innovation Macquarie University Sydney New South Wales Australia; ^2^ Consumer Representative; ^3^ Faculty of Medicine, Nursing and Health Sciences Monash University Melbourne Victoria Australia; ^4^ Safer Care Victoria Melbourne Victoria Australia; ^5^ University of New South Wales Sydney New South Wales Australia

**Keywords:** adverse events, apology, communication, open disclosure, patient experience, patient safety, Statutory Duty of Candour, trust

## Abstract

**Background:**

The Statutory Duty of Candour (SDC) legally requires healthcare organisations to notify patients, families, and carers who experience a serious adverse patient safety event (SAPSE), provide an apology, and explain steps taken to prevent recurrence. However, despite growing international adoption of SDC, evidence on its implementation and impact on patients and families (‘consumers’) remains limited. This study examined consumer awareness, understanding, and lived experiences of SDC in Victoria, Australia, two years after its introduction.

**Methods:**

A mixed‐methods study integrated data from an online survey and semi‐structured interviews with healthcare consumers in Victoria, Australia. Descriptive analysis was undertaken for quantitative items, while reflexive thematic analysis was applied to qualitative data from survey free‐text responses and interview transcripts.

**Results:**

In total, 80 consumers participated: 72 completed the survey and 13 completed interviews, with five participating in both. Although nearly two‐thirds (61%) had no prior knowledge of SDC, over three‐quarters (78%) perceived it as useful. Among those who had experienced an adverse event, half reported that staff were not open following the incident, over half reported that no post‐incident meeting occurred, and satisfaction with meetings was low (26%). Six themes were identified: (1) Candour as Moral Reassurance; (2) Persistent Scepticism and Conditional Trust; (3) Limited Awareness and Accessibility of SDC; (4) Genuine Apology for Trust Repair; (5) Emotional Labour, Power, and the Risk of Re‐traumatisation; and (6) Demonstrating Accountability with Organisational Learning and Improvement. An overarching theme, *Candour as a Relational Process for Trust,* described how trust was tested, repaired, or further eroded through SDC processes following an adverse event.

**Conclusion:**

SDC holds significant potential to repair trust following adverse events, but its impact depends on how it is enacted. When delivered with empathy and meaningful follow‐up, SDC supports repair of trust. However, when experienced as procedural or defensive, SDC may compound distress and further erode trust. Therefore, SDC must be embedded not just as a regulatory requirement, but as a relational and person‐centred practice for patients and families.

**Patient or Public Contribution:**

Healthcare consumer representatives were part of the research team, assisting with the study design, data analysis, and article writing.

## 1 Introduction

When patients are harmed during healthcare, the consequences extend far beyond the immediate clinical event, with physical injury often accompanied by significant emotional, psychological, and financial distress for patients and families [1]. However, research shows that it is the response to harm, particularly the quality of communication, that most strongly shapes patients’ long‐term experiences of trust and recovery [2]. Poor communication, avoidance, or defensiveness by healthcare professionals and organisations can compound trauma [3, 4]. In contrast, honesty, compassion, and timely communication are associated with greater satisfaction, reduced distress, and sustained trust in clinicians [5, 6].

Globally, healthcare systems have introduced policies to support openness and apology following adverse events. In the United States, disclosure and apology programs, and Communication and Resolution Programs (CRPs), sought to promote transparency, learning, and reconciliation while reducing liability exposure [2, 7]. Similarly, the United Kingdom's *Being Open* framework and Australia's *Open Disclosure Framework* aimed to ensure patients and families are informed when harm occurs, and receive an apology and explanation [8, 9]. Yet, despite these policy advances, implementation of open disclosure has been inconsistent. Studies have identified wide variability in how clinicians and organisations communicate after harm, with patients often reporting delayed or incomplete disclosure, absent apology, and limited follow‐up or support [10, 11, 12]. Open communication may be inhibited by organisational cultures of blame and staff uncertainty, including fear of litigation [7, 13, 14, 15].

Accordingly, to strengthen healthcare accountability and improve consistency of disclosure processes, several jurisdictions have legislated candour as a legal duty of healthcare organisations. The *Statutory Duty of Candour (SDC)* was first introduced in the UK National Health Service in 2014 in response to major inquiries into failures of openness and adverse safety events [16]. The legislation requires organisations to notify patients of adverse events causing harm, provide an apology, and explain actions taken to prevent recurrence [17, 18]. Notably, this shift marked an important development from professional and ethical responsibility toward formal legal requirements for healthcare organisations [19]. Since then, similar frameworks have been adopted internationally, with varying thresholds for inclusion and mechanisms for enforcement. In Victoria, Australia, the SDC was implemented in 2022 through the *Victorian Health Legislation Amendment (Quality and Safety) Act* [20]. The legislation forms part of a broader cultural reform agenda led by *Safer Care Victoria (SCV)*, the state's peak agency for healthcare quality and safety. Under the Act, when a patient experiences a Serious Adverse Patient Safety Event (SAPSE), the health service is legally required to implement the SDC process.

Furthermore, SDC aligns with principles of *restorative justice*, which highlights that an effective apology requires both an acknowledgement of responsibility and an offer of repair [21]. Accordingly, SDC must involve clear ownership of harm and visible actions to address its consequences and repair damaged relationships, which is particularly important given the complex and enduring nature of harm following adverse events. Patients and family members have reported long‐term and secondary harms for years after the original event [22].

Although SDC policy is being increasingly established in healthcare systems, there is limited research regarding its implementation or its impact on patients, families, staff, and health services. Our recent systematic review highlighted a lack of published evaluations of statutory or professional candour obligations and their influence on trust, learning, and patient experience [18]. Although a review of duty of candour policy is currently underway in the UK, no research has yet been published to evaluate SDC implementation or outcomes [23]. To address this gap, the Victorian Government commissioned an independent evaluation of SDC implementation, conducted by a team of consumers, clinicians, and academic researchers in partnership with SCV. Collectively, this research seeks to understand how SDC has been enacted in practice and perceived by patients, families, and healthcare staff. As part of a broader evaluation [24], this paper reports on one component of a larger mixed‐methods study exploring patient and clinician perspectives of SDC in Victoria, Australia.

## 2 Aim

The aim of this study was to explore consumer perspectives of the Statutory Duty of Candour in Victoria, Australia, including both general perceptions and lived experiences of participation in SDC processes. The specific objectives were:

1. To examine the level of awareness, knowledge, and understanding of SDC among Victorian health consumers;
2. To explore the experiences, perceived effectiveness, and impact of SDC among patients and families who had participated in an SDC process, including perceived barriers and enablers to its implementation.

## 3 Methods

### 3.1 Design

This mixed‐methods study integrated survey data and semi‐structured interviews from patients and healthcare consumers. Survey data offered indicative patterns of awareness and perception about SDC, while qualitative data provided depth and contextual understanding to expand on the quantitative findings, including consumer experiences of the SDC process.

### 3.2 Setting and Participants

Participants were adult healthcare consumers (aged 18 years and older) residing in Victoria and accessing health services in the state. Consumers were eligible if they had received care within Victorian health services, or supported a family member who had, since the introduction of SDC (i.e. in the last 2 years). In this study, the term *consumer* is used in preference to *patient* to reflect the conventions of the Victorian health system and broader Australian health policy, which use *consumer* to refer to people who use health services, including patients, carers, and family members [25]. This differs from terminology commonly used in the United Kingdom and United States, where *patient* is more prevalent. The terms are used interchangeably in some instances to reflect the language used by participants themselves and in the broader literature.

Participants included both *general consumers* to explore awareness and perceptions of SDC, and *consumers with lived experience* who had participated in the SDC process, including patients and/or their family members. Multiple channels were used for project recruitment to ensure diversity of perspectives, including recruitment posters in newsletters from *Health Voices Victoria* (a health consumer network), *Safer Care Victoria* consumer registries, university‐affiliated consumer networks, and professional networks (e.g., LinkedIn). Participants could contribute through an anonymous online survey and/or a confidential semi‐structured interview.

### 3.3 Data Collection

Data were collected for a 6‐month period (between February 2025 and August 2025), approximately 2 years after the implementation of SDC in Victoria, Australia.

### 3.4 Survey

The survey was adapted from previously used instruments to assess open disclosure policy implementation in the UK [26], with items contextualised to the Australian setting by consumers and service providers on the research team. The final survey included both closed and open‐ended questions exploring: (a) awareness, knowledge, and understanding of SDC, and (b) perceived effectiveness and impact of SDC on the management of patient safety events. The survey was administered via Qualtrics software, with an anonymous link embedded in all recruitment materials. Participants could complete the survey independently and, at its conclusion, opt to participate in a follow‐up interview by providing contact details or emailing the research team directly.

### 3.5 Interviews

Semi‐structured interviews were conducted by an experienced qualitative researcher (CA) with expertise in patient experience and safety research. Interviews lasted for 30‐45 min. The interview guide explored participants’ awareness and understanding of SDC, experiences of communication following adverse events, and reflections on apology, accountability, and organisational learning. Interviews were conducted online via Microsoft Teams to facilitate participation across the state of Victoria. With participant consent, all interviews were audio‐recorded and transcribed verbatim. Transcripts were de‐identified and stored securely on Macquarie University's OneDrive in accordance with institutional data protection protocols.

### 3.6 Data Analysis

#### 3.6.1 Quantitative Analysis

Quantitative data from closed survey items were analysed using R (version 4.2.2). Frequencies and proportions were calculated for awareness, perceived impact, and satisfaction variables. Levels of agreement on these factors were depicted using five‐point Likert scales. Quantitative findings were used to contextualise qualitative themes and enhance interpretive triangulation.

#### 3.6.2 Qualitative Analysis

Free‐text survey responses and interview transcripts were analysed together to provide a comprehensive understanding of consumer views, which acknowledged that some participants preferred written anonymity when discussing sensitive experiences (such as harm and adverse events). Qualitative data were analysed using reflexive thematic analysis [27], managed in NVivo 12 (QSR International, Melbourne, Australia). Two researchers (LB and MB) and a consumer representative (JM) supported the reflexive thematic analysis process, reviewing the transcripts and coding the data. Initial themes were generated with another researcher (CA), while the full authorship team reviewed and agreed the final themes. The research team included clinicians from nursing, pharmacy and psychology, patient experience and quality and safety researchers, and members affiliated with Safer Care Victoria and/or the commissioning body. Reflexive discussions during coding and theme development considered how these clinical, policy and organisational perspectives shaped interpretation, while supporting close engagement with participants’ accounts.

Mixed‐methods integration was undertaken using a complementary approach, whereby survey findings were used to identify broad patterns in consumer perceptions of the Statutory Duty of Candour, while qualitative findings were used to contextualise, deepen and explain these patterns through participants’ accounts of expectations, concerns and lived experiences.

### 3.7 Ethical Considerations

Ethics approval was granted by the Human Research Ethics Committee at Macquarie University (Project ID 520241807758893). Participation was voluntary, and participants were informed that reflecting on healthcare harm could evoke emotional distress. Completion of the online survey implied consent, while written informed consent was obtained for all interviews. Participants were provided with information on psychological support to ensure their safety and wellbeing throughout the research process.

## 4 Results

A total of 80 consumers participated in the study, with 72 completing the survey and 13 completing semi‐structured interviews. Five consumers completed both the survey and interview.

### 4.1 Survey Findings

In total, 72 consumers completed one or more of the survey questions. The demographic characteristics of respondents are shown in Table 1, with just over two‐thirds of respondents being female; the largest age group was 46–65 years (44%).

**Table 1:** Demographic characteristics of survey respondents.

Respondent characteristics	*n* * (%)
**Age group**	
18–45 years	20 (27.8%)
46–65 years	32 (44.4%)
≥ 66 years	11 (15.3%)
Unspecified	9 (12.5%)
**Gender**	
Female	49 (68.1%)
Male	13 (18.1%)
Unspecified	10 (13.9%)

* Results are reported for respondents who answered each item.

Table 2 provides responses to items that explored consumer perceptions and experiences of SDC. Overall, although many respondents (61%) reported no prior knowledge of SDC, over three‐quarters (78%) perceived SDC to be useful to inform patients about adverse events. Among the 58 respondents who answered this item, more than half (59%) reported experiencing an adverse patient safety event (APSE), which primarily occurred in public hospitals (74%). Of those who experienced an APSE, approximately half (53%) believed their harm to be major or severe, with most (62%) having self‐identified the incident. There were mixed responses about the perceived openness of healthcare staff, occurrence of post‐incident meetings, and satisfaction with the meetings.

**Table 2:** Consumer perspectives and experiences related to Adverse Patient Safety Events and the Statutory Duty of Candour.

	n* (%)
**Prior awareness and knowledge about SDC**	
No	44/72 (61.1%)
Yes	28/72 (38.9%)
**Perceived usefulness of SDC in informing patients about adverse events**	
Yes	56/72 (77.8%)
No/Unsure	16/72 (22.2%)
**Self‐reported experience of an Adverse Patient Safety Event (APSE)**	
No	24/58 (41.4%)
Yes	34/58 (58.6%)
**Setting of APSE**	
Public Hospital	25/34 (73.5%)
Private hospital/Other	9/34 (26.5%)
**Experience of harm resulting from an APSE**	
No harm	9/34 (26.5%)
Minor harm	7/34 (20.6%)
Major/Severe harm	18/34 (52.9%)
**Timing of incident awareness**	
During/Immediately	17/33 (51.5%)
Within 7 days	10/33 (30.3%)
After 7 days/Not sure	6/33 (18.2%)
**Who first identified the incident**	
Self	21/34 (61.8%)
Someone else (Healthcare staff, family, carer)	13/34 (38.2%)
**Perceived openness of healthcare staff**	
No	17/34 (50.0%)
Yes	11/34 (32.4%)
Not sure	6/34 (17.6%)
**Post‐incident meeting with healthcare staff**	
No	18/33 (54.5%)
Yes	15/33 (45.5%)
**Satisfaction with timeliness of staff communication about the incident**	
No/Unsure	13/20 (65.0%)
Yes	7/20 (35.0%)
**Satisfaction with the SDC or open disclosure meeting with healthcare staff**	
No	17/31 (54.8%)
Yes	8/31 (25.8%)
Unsure	6/31 (19.4%)

**Figure 1:**
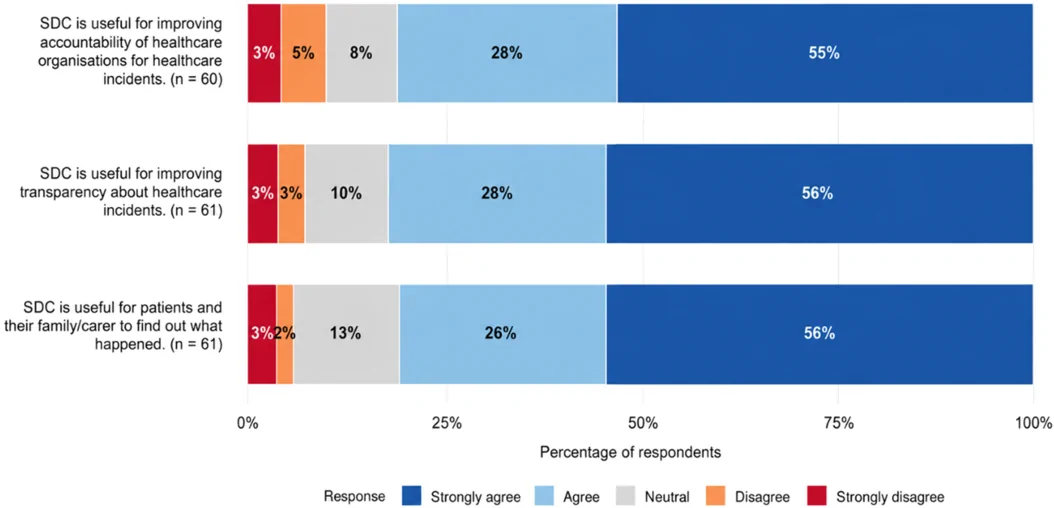
Consumer perceptions about the usefulness of Statutory Duty of Candour.

Findings from the survey items about consumer perceptions about the usefulness of SDC regarding transparency, accountability, and communication with patients and family members are shown in Figure 1.

### 4.2 Qualitative Themes

Six themes were identified regarding consumer perspectives about SDC: *(1) Candour as Moral Reassurance; (2) Persistent Scepticism and Conditional Trust; (3) Limited Awareness and Accessibility of SDC; (4) Genuine Apology for Trust Repair; (5) Emotional Labour, Power, and the Risk of Re‐traumatisation; and (6) Demonstrating Accountability with Organisational Learning and Improvement*. An overarching theme was generated from the free‐text survey responses and semi‐structured interviews: *Candour as a Relational Process for Trust*.

The six themes reflect a progression from how SDC is understood in principle to how it is experienced in practice, and should be read in relation to one another. Themes 1 and 2 draw on the perspectives of general consumers, capturing the tension between broad support for candour as a moral right and scepticism about its genuine enactment. Theme 3 identifies structural barriers that can limit equitable access to SDC before it is initiated, including low awareness, legalistic language, and activation thresholds. Themes 4 and 5 draw on accounts from consumers with lived experience of SDC, examining the relational quality of the encounter itself and how apology sincerity, emotional labour, and power dynamics shape whether candour repairs or further erodes trust. Theme 6 moves beyond the formal encounter to address whether SDC fulfils its deeper purpose through organisational learning, consumer follow‐up, and quality improvement.

**Theme 1: Candour as Moral Reassurance**

For participants who had not personally experienced the SDC process, the policy was understood as a form of moral assurance within the healthcare system. Consumers described feeling reassured about the existence of a legislated mandate for openness, which was interpreted as a safeguard against concealment, silence, or minimisation of harm. SDC was seen as an indicator that honesty and accountability were formally valued by the healthcare system, providing a sense of protection and transparency from healthcare providers.

Even if no mistakes are made, it would be reassuring to know processes like this are in place.

(Cons41)

It gives that kind of safety net behind you… people have a right to find out why it's gone wrong.

(Cons11)

In this sense, SDC functioned as a symbolic guarantee of ethical conduct. Consumers described the policy as providing reassurance that, should harm occur, patients and families would be entitled to truthful information, acknowledgement, and explanation. This symbolic function was particularly important in shaping baseline trust in healthcare institutions, even in the absence of direct experience with SDC. As such, it was seen as an essential component of a trustworthy health system that upholds both justice and respect following adverse events.

It makes you feel empowered, like you've got rights in how you're cared for.

(Cons10)

Therefore, candour was understood as a moral and procedural right, closely linked to expectations of fairness, honesty, and respect following adverse events. SDC was seen to formalise what should already be an inherent aspect of clinical professionalism, with honest and compassionate communication following harm. For many participants, SDC was therefore viewed as both reassuring and necessary, a formalisation of moral expectations that have not always been reliably upheld in practice. Importantly, this theme highlights that trust in SDC often preceded trust in its implementation. Consumers expressed confidence in the *idea* of candour, even while questioning whether it would be enacted authentically in practice.

Great concept. Would like to see it applied in real life.

(Cons41)

**Theme 2: Persistent Scepticism and Conditional Trust**

While participants expressed strong conceptual support for candour as moral assurance and a patient right (Theme 1), this support was often accompanied by fragile trust and scepticism about how the SDC would be enacted in practice. Consumers questioned whether legislating candour could produce genuine openness, or whether it risked becoming a procedural exercise driven by organisational self‐protection rather than moral accountability.

A formal apology should be made separately, not just because the SDC says to apologise.

(Cons49)

I don't know that an apology can be legally mandated. It wouldn't feel genuine.

(Cons57)

Scepticism was often informed by prior experiences of defensiveness within healthcare systems, leading participants to doubt whether SDC would meaningfully shift entrenched organisational behaviours. Several participants reported that health services prioritised reputation management and legal risk over transparency and consumer needs.

I hope it works, but I have doubts. Hospitals don't like admitting fault when harm's been done.

(Cons28)

Hospitals are all about protecting the brand above client care.

(Cons12)

The process is for the service, not for the family/consumer.

(Cons7)

This scepticism was further reinforced by concerns about who controls access to candour, particularly due to the thresholds for SDC activation. First, they noted that the definition of a ‘serious adverse patient safety event’ focuses mainly on physical harm, while psychological and emotional harms are often overlooked in practice, despite being recognised in related guidelines. Second, participants questioned the expectation that harm must also be considered ‘severe’ by the health service before SDC is triggered.

What they [clinicians] might interpret as a moderate or severe might be different to patient and carers and family. Obviously, the physical ones are easier to detect and categorise. But sometimes mental scarring, or even trauma from a negative incident, may have a huge impact on the patient and the carers.

(Cons5)

It seems to only apply when very major incidents happen. But nothing exists for lesser issues.

(Cons29)

It needs to include a process for not so serious incidents of harm. That is not too onerous, but satisfies the needs of the harmed patient.

(Cons36)

Together, these concerns suggest that SDC may prioritise organisational and clinical judgments over patients’ own understandings of harm. As a result, some patients may experience harm that feels significant to them but does not meet institutional thresholds for SAPSE. Ultimately, this may limit their access to SDC review processes. Although scepticism was common, consumers expressed cautious optimism that genuine and empathetic SDC processes could promote trust, accountability, and improvement. However, the gap between the ethical intent of SDC and its potential bureaucratic implementation produced a fragile form of trust, which was conditional not solely on the legislation itself but on the quality of health services’ enactment of it.

**Theme 3: Limited Awareness and Accessibility of SDC**

Despite strong support for the principles of candour, awareness of SDC was low, including among participants who had experienced adverse safety events.

Patients and families need to know the SDC exists. This is the first I've heard of it and my knowledge of the health system is very good.

(Cons46)

It would have been beneficial if I was told at the time about this by hospital staff. But I wasn't, sadly.

(Cons27)

Knowing that candour processes existed, and understanding what they involved, was seen as essential for patients and families to feel informed, empowered, and able to engage confidently following harm. In the absence of such awareness, candour was perceived as something controlled by the organisation, rather than a right that is available to all healthcare consumers.

It gives you comfort knowing the process is there. But they should explain it upfront, before anything goes wrong.

(Cons5)

Communicate to patients that it actually exists… And that we have rights as healthcare consumers to this kind of information and accountability.

(Cons41)

Several consumers suggested that proactive communication about SDC is necessary, including visible information in hospitals, such as posters about SDC, and inclusion of SDC information in hospital admission materials.

Educate the community about what it is and how it will work.

(Cons49)

Have information displayed about it openly in hospitals.

(Cons48)

Consumers also identified language and terminology as a barrier to access for SDC. The term ‘*Statutory Duty of Candour’* was widely described as overly legalistic, bureaucratic, and misaligned with the emotional and relational nature of harm experiences. This terminology created unnecessary complexity that conflicted with the intention of promoting clear and open communication, and was seen as potentially exclusionary for consumers with lower health literacy and/or from culturally and linguistically diverse (CALD) backgrounds.

Please create a simple, clear name instead of this difficult one.

(Cons40)

Less corporate speak. More compassion!

(Cons38)

Simplify the language. The name ‘Statutory Duty of Candour’ is far too complex for the health literacy of many health consumers.

(Cons25)

Collectively, these findings suggest that the combination of legal terminology and limited public information may create a perception that SDC is primarily a legal or procedural requirement. When candour is framed (or perceived) in primarily legal or procedural language, it risks reinforcing power asymmetries and positioning patients as passive recipients, rather than active participants in safety and accountability processes. Hence, participants emphasised that, for candour to feel authentic and meaningful, it must be communicated in language that is clear, accessible, and aligned with the needs of those it is intended to support.

**Theme 4: Genuine Apology for Trust Repair**

For consumers who had engaged directly in the SDC process, the quality and sincerity of the apology emerged as central to whether trust could be repaired following harm. Participants highlighted a distinction between apologies delivered with sincerity and genuine moral accountability, and those perceived as tokenistic and procedural requirements. Genuine apologies by healthcare providers were characterised by humility, empathy, and explicit acknowledgement of organisational responsibility, rather than adherence to scripted or legally‐framed responses.

We felt genuine care from the [medical] team. They were disappointed by what happened and genuinely didn't want it to happen again.

(Cons7)

How important it is just to say sorry… It's validating and you feel you can trust a little bit more, even if they made a mistake.

(Cons7)

Apologies perceived as sincere were described as relationally reparative, validating patients’ and family members’ experiences. Consumers reported valuing apologies and expressions of regret that were accompanied by attentive listening, clear explanations of what had occurred, and acknowledgement of organisational responsibility. In these instances, apology functioned as a mechanism for rebuilding trust and restoring a sense of human connection. Conversely, when apologies were perceived as formulaic, defensive, or scripted, they were interpreted as acts of organisational self‐protection.

It was very formal, like they were just getting through it. That didn't help at all.

(Cons8)

Consumers described such encounters as superficial and disengaging, often reinforcing scepticism about organisational motives and further eroding trust. As such, apology within SDC was not experienced as a discrete communicative act, but as part of a broader relational process of moral repair.

**Theme 5: Emotional Labour, Power, and the Risk of Re‐traumatisation**

Participants who had undergone SDC described mixed experiences about the emotional toll of the process. For some, the SDC process was very positive, helping to support understanding, validation, and closure.

I feel grateful and lucky to have had the opportunity to start to get that closure to work through the event myself… to move on from the negative feelings of shame and guilt and all of that.

(Cons10)

However, for others, the SDC process was described as emotionally demanding, particularly when they were already coping with grief, ongoing injury, or long‐term health consequences. As such, recalling and discussing adverse events during the SDC process could trigger renewed distress, anger, and frustration in patients and family members.

You relive the memories again. You've got to go through it again and again. It's bloody hard.

(Cons8)

My husband found this [SDC] exhausting on top of his medical journey.

(Cons7)

An important feature of this emotional labour was the perception that the responsibility for initiating and sustaining candour frequently fell to patients and families themselves. Several participants reported that SDC processes were activated only after they lodged formal complaints, rather than being proactively initiated by the healthcare system. As such, this transferred significant emotional and administrative labour on to those already harmed.

The hospital should approach the consumer to organise open disclosure, not be chased.

(Cons30)

Several participants also described SDC meetings as intimidating, often characterised by procedural formality and clinical hierarchy. In some instances, the presence of multiple senior staff members was perceived as reinforcing power imbalances, limiting opportunities for open and equitable dialogue during SDC meetings.

They stack the table with every doctor you have seen, even when they aren't involved. This was a power trip. We are bigger than you.

(Cons22)

Just purely because of their power, they made me feel like I was not credible.

(Cons12)

Rather than fostering partnership, these encounters at times reproduced existing power asymmetries within healthcare, thus undermining the relational intent of candour. The emotional labour required of patients and families, coupled with the administrative complexity of the SDC process, left some participants feeling further disempowered rather than supported. These experiences highlight the risk of re‐traumatisation when candour is poorly enacted or insufficiently supported. Notably, when emotional labour is shifted onto patients and families, and power imbalances remain unacknowledged, candour may move from a mechanism intended to support trust and healing to one that inadvertently compounds harm and distress.

**Theme 6: Demonstrating Accountability with Organisational Learning and Improvement**

Most participants noted that their motivation for being involved in the SDC process was to help others and prevent reoccurrence of similar adverse events. Hence, this sense of contributing to positive change facilitated closure for some consumers, providing reassurance that something meaningful emerged from their experience.

I don't want to see another family go through the heartache and emotions I've had to go through.

(Cons8)

It was worth the effort and will hopefully help someone else down the track.

(Cons6)

There's the sadness and anger that this event happened to me. But there's another part of me where I want to make sure that this event is avoided in future for other patients, other mothers, other babies. I guess it's about doing some good out of a bad situation for me, as well as for the hospital, if they can.

(Cons10)

A recurring expectation was that the SDC process would lead to organisational learning and tangible improvements in quality and safety. Participants who received updates about subsequent improvements and saw evidence of action found the SDC process more credible, and expressed greater satisfaction and empowerment from their contribution.

There was open discussion about the error and safeguards in place to prevent it. That gave me confidence.

(Cons35)

I actually feel really empowered that I was able to provide input into how things can change and what can change.

(Cons11)

Conversely, when there was no follow‐up or visible healthcare improvement, participants expressed frustration and perceived SDC as merely a compliance exercise. The absence of visible improvement created perceived *lack of consequence*, a symbolic acknowledgement of harm that was unaccompanied by institutional learning or improvement.

It was a very standard response that, at the end of the day, has not addressed the situation.

(Cons34)

I feel as though what we said wasn't taken on board.

(Cons8)

Another important element was the desire for justice and accountability. While participants acknowledged that mistakes could happen, they expected organisations to acknowledge errors and take responsibility for their mistakes.

Someone made a mistake. I'd prefer they just said that.

(Cons7)

It would've made it a lot easier to swallow that they'd stuffed up and like, yes, everybody's human. Yes, everybody makes mistakes.

(Cons8)

Even when people make mistakes, if people have just been honest at the start and taken accountability for it, then it wouldn't have caused the harm it caused.

(Cons12)

Notably, participants reported that the formal SDC process did not address all of their needs, particularly regarding ongoing communication and alternative supports. Ongoing contact beyond the timeframes set for SDC was viewed as a sign of respect and commitment to change. In contrast, when the SDC process ended abruptly, some participants felt abandoned.

There's been no follow‐up after that. It's like they've ticked the box of the review… If there's an ongoing injury or a potentially long‐term or permanent injury, there needs to be more of a follow‐up from the review. Not just finish there, and that's it.

(Cons7)

In addition to information and learning, participants described unmet needs for emotional and practical support, including counselling, welfare checks, and assistance with home care. The absence of such support was perceived as a failure to acknowledge the enduring consequences of harm.

There was no welfare check on the family to make sure they were okay. This [incident] happened under their watch. They did nothing to follow up with the family apart from saying ‘we want to have a meeting with you’.

(Cons8)

There was no offer of practical support, such as housecleaning, meal vouchers. An apology is nice, but what is needed is practical support.

(Cons7)

**Overarching Theme: Candour as a Relational Process for Trust**

Across all participant accounts, trust emerged as both the foundation and the measure of candour. SDC was interpreted as a moral promise: a commitment that when harm occurs, patients and families will be met with honesty, accountability, and care.

People trust the health system with their lives, and with their family's lives.

(Cons10)

Consumers experienced SDC as a relational process through which trust was tested, repaired, or further eroded. The perceived success or failure of SDC depended not only on compliance with legislative steps, but on how those steps were enacted. Trust was strengthened when candour was delivered with empathy and genuine accountability, and undermined when interactions were perceived as defensive, scripted, or institution‐centred.

Getting that response from her made me think, okay, someone's on the ball. I can start to trust that service again. For a patient to trust the service is the most important thing.

(Cons6)

We'd walk out of meetings and say, they're covering their ass. They're doing this for themselves, not for us.

(Cons8)

Participants described trust as both the precondition and outcome of candour. Apology alone was rarely sufficient to restore it, with visible learning, follow‐up, and genuine accountability equally essential. As such, procedural adherence without relational engagement was consistently interpreted as symbolic compliance rather than moral accountability. Collectively, these findings reframe SDC not as a time‐limited legislative obligation, but as an ongoing relational practice – one in which trust must be actively built and carefully sustained, including after the formal SDC process concludes.

## 5 Discussion

This study provides the first in‐depth examination of patient and consumer perspectives on the Statutory Duty of Candour (SDC) in Australia during its early implementation. From the survey results, over three‐quarters of respondents (78%) perceived SDC as useful; however, nearly two‐thirds (61%) reported no prior knowledge of its existence. Given that recruitment likely overrepresented highly engaged consumers, the true extent of public unawareness may be even greater. These findings suggest several opportunities to refine SDC implementation, including simplifying the legislation's title to improve accessibility, and introducing greater flexibility in procedural timeframes to better support person‐centred care.

Notably, trust emerged as the central mechanism through which SDC was interpreted and evaluated. Consumers reported that SDC symbolised a promise of transparency, apology, and repair; however, this trust was fragile and easily undermined. This fragility is reflected in the survey data: half of respondents who experienced an adverse event (50%) perceived healthcare staff as not open following the incident, and more than half (55%) reported that no post‐incident meeting occurred. When candour was enacted with empathy, humility, and meaningful follow‐up, trust could be restored. When interactions were perceived as defensive, scripted, or procedurally driven, trust was further eroded. These findings build upon international research showing that candour is not experienced as a single disclosure event but as an ongoing, relational process of trust repair, shaped by sincerity, power dynamics, emotional labour, and visible organisational accountability [1, 2, 28]. They also align with New Zealand health policy emphasising healing from harm as a relational process, with the stated goal of addressing harms, meeting needs, restoring trust, and promoting healing for all involved [28].

A key contribution of this study is the distinction it illuminates between procedural and relational candour. Although participants broadly endorsed the principle of candour, many expressed scepticism about whether it would be enacted with genuine accountability rather than institutional self‐protection, thereby reflecting concerns in the literature regarding legally driven disclosure versus morally grounded candour [2, 19]. This is reinforced by survey findings: among those who attended a post‐incident meeting, over half (55%) reported dissatisfaction, and 65% rated the timeliness of staff communication as inadequate or uncertain. Participants distinguished between apologies perceived as genuine and compassionate, and those experienced as performative and tokenistic. Consistent with existing research, effective candour required both cognitive components (such as clear explanations and acknowledgement of responsibility) and affective components, including empathy and emotional engagement [6, 28, 29]. If these elements were absent, candour may be perceived as inadequate, and could be further damaging for patients and families.

These findings also challenge the positioning of clinicians and organisations as sole holders of knowledge, with patients framed as passive recipients of information [30]. Survey data indicated that 62% of respondents who experienced an adverse event had self‐identified the harm, rather than learning of it through disclosure by the health service, aligning with research demonstrating that patients and families are active sense‐makers with valuable experiential knowledge [1, 10, 31]. This raises important questions about organisational thresholds for ‘serious harm,’ particularly where psychological and emotional harms are experienced as profound by consumers but may not trigger formal SDC processes. The significance of emotional burden within the SDC process is underscored by the survey data: over half of those who experienced an adverse event (53%) considered their harm to be major or severe, yet only one‐quarter (26%) reported satisfaction with post‐incident meetings. In this study, power imbalances, intimidating meeting structures, and the repeated recounting of traumatic events rendered the SDC process emotionally exhausting for many participants. Hence, without attention to emotional vulnerability and power dynamics, candour processes risk re‐traumatising patients and families rather than supporting healing [32, 33]. These findings highlight the importance of trauma‐informed approaches to SDC enactment, which foreground emotional safety, empowerment, and trust‐building as preconditions for genuine candour.

From a restorative justice perspective, participants expected candour to involve more than apology; they wanted accountability, learning, and visible system improvement [22]. Therefore, SDC processes require demonstrable action to prevent recurrence, which may extend well beyond the timeframe of the formal disclosure process. Research also suggests that this healing is mutual: structured post‐incident support can assist recovery for both patients and healthcare workers [34], reinforcing the relational and bidirectional nature of trust repair after harm. Collectively, principles drawn from relational ethics, trauma‐informed care, and restorative justice explain why consumers experience candour not as a discrete event, but as an ongoing process requiring emotional safety, accountability, and meaningful action.

Overall, these findings affirm that SDC holds substantial potential to strengthen trust, accountability, and learning following harm. Yet, this can only occur when SDC is enacted as a relational practice, rather than a procedural obligation. The gap between legislative intent and lived experience, evident throughout this study, signals that statutory compliance alone is insufficient to achieve meaningful candour. Therefore, for SDC to fulfil its intended purpose, healthcare organisations must move beyond the mechanics of disclosure toward a sustained commitment to empathetic communication, recognition of patient knowledge, emotional support, and visible organisational learning. When these conditions are met, SDC can achieve its deeper purpose: not merely to ensure that patients are informed when harm occurs, but to genuinely repair the trust that harm has broken.

### 5.1 Practical, Policy and Research Implications

For SDC to achieve its intended impact, legal requirements must be translated into relational capabilities that support trust repair and emotional care. Clinician training should extend beyond procedural knowledge to include empathy‐based communication and skills for managing challenging conversations. At an organisational level, infrastructure must support relational candour through adequate time, supervision, and debriefing opportunities. Emotional and practical support for patients and families could be strengthened through dedicated roles, such as patient advocates, or counselling services — as implemented through New South Wales’ dedicated family contact model following serious adverse safety events [35]. Importantly, post‐incident support should also extend to healthcare workers involved in adverse events, given evidence that mutual healing processes benefit both patients and clinicians [36, 37].

Public awareness of SDC requires significant improvement. Clear, accessible communication about patient rights and candour processes may empower consumers to engage more equitably in safety dialogues. Furthermore, greater alignment between SDC, Open Disclosure, and complaints management processes would also reduce fragmentation and improve continuity of support. Policy refinement is needed to ensure SDC responds to the full range of consumer‐experienced harms, particularly psychological and emotional harm, and a review of SAPSE thresholds may help align SDC policy more closely with consumer expectations. Future research should examine factors that support trust repair within SDC processes, including longitudinal studies assessing the sustained impact on patient–provider relationships and complaints patterns. Further work should prioritise the experiences of culturally and linguistically diverse (CALD), and remote and rural communities, and explore the role of leadership, organisational culture, and clinician wellbeing in SDC implementation.

### 5.2 Strengths and Limitations

This study has several important strengths. It is the first published examination of consumer perspectives on SDC in Australia, and one of the first internationally, addressing a significant evidence gap during early policy implementation. The mixed‐methods design enabled both breadth and depth of understanding, while the integration of consumer representatives throughout, including in study design, data analysis, and manuscript development, strengthens the credibility and consumer‐centredness of the findings. The recruitment of both general consumers and those with lived experience of SDC enabled perspectives to be captured across the full spectrum of consumer engagement with the legislation.

The findings should be interpreted in light of several limitations. Individuals with strong views about SDC may have been more likely to participate, which may limit transferability. The study was conducted during early implementation in Victoria, and findings may reflect transitional practices that evolve over time or differ in other contexts. No data were collected on consumer demographics such as cultural or linguistic diversity or geographic location, which are important dimensions for future research. Finally, as awareness of SDC as a distinct legislative mechanism was low, some participants may have conflated SDC‐specific experiences with broader open disclosure or complaints processes.

## 6 Conclusion

Patient and consumer perspectives during the early implementation of the Statutory Duty of Candour (SDC) indicate that candour is experienced not as a single disclosure event, but as an ongoing process that shapes trust after harm. While participants strongly supported the principle of SDC, its impact depended on how it was enacted in practice. When communication was sincere, empathetic, and followed by meaningful action, candour supported accountability and trust repair. When it was experienced as procedural or defensive, it often increased distress and undermined confidence in healthcare organisations. Overall, these findings suggest that SDC has significant potential to strengthen trust, learning, and accountability in healthcare systems. However, realising this potential requires moving beyond compliance‐based approaches and embedding candour as a *relational practice of trust repair*, which includes recognising patient and family knowledge, attending to emotional safety, and ensuring visible organisational learning and follow‐up. As SDC continues to mature, prioritising these relational elements offers a clear opportunity to translate legislative intent into more meaningful and person‐centred responses to harm in healthcare.

## Author Contributions

**Corey Adams:** writing – original draft, methodology, writing – review and editing, formal analysis, project administration, conceptualisation. **Lorelle Bowditch:** writing – review and editing, formal analysis, investigation. **Mia Bierbaum:** investigation, writing – review and editing, formal analysis. **Ramya Walsan:** writing – review and editing, software, formal analysis, data curation. **Jennifer Morris:** methodology, writing – review and editing, validation. **Elizabeth Manias:** methodology, writing – review and editing, supervision. **Nicole Youngs:** writing – review and editing, validation, methodology, project administration. **Lanii Birks:** writing – review and editing, validation. **Jeffrey Braithwaite:** supervision, writing – review and editing, conceptualisation, methodology. **Ashfaq Chauhan:** formal analysis, writing – review and editing, methodology. **Peter Hibbert:** methodology, supervision, formal analysis, conceptualisation, investigation, writing – review and editing. **Ramesh Walpola:** writing – review and editing, formal analysis, supervision, methodology. **Reema Harrison:** supervision, project administration, funding acquisition, methodology, writing – review and editing, formal analysis, conceptualisation.

## Ethics Statement

Ethics approval was granted by the Human Research Ethics Committee at Macquarie University (Project ID 520241807758893).

## Conflicts of Interest

N.Y. and L.B., are employed by Safer Care Victoria, which commissioned and funded this evaluation. All analyses and interpretations were undertaken independently, and the views expressed in this article are those of the authors alone.

## Supporting information


Supporting File

## Data Availability

The data that support the findings of this study are not publicly available due to ethical restrictions and the sensitive nature of the data, which include personal experiences of harm in healthcare. De‐identified data may be available from the corresponding author upon reasonable request, subject to ethics approval and data sharing agreements.

## References

[1] R. Iedema , R. Sorensen , E. Manias , et al., “Patients’ and Family Members’ Experiences of Open Disclosure Following Adverse Events,” International Journal for Quality in Health Care: Journal of the International Society for Quality in Health Care 20, no. 6 (2008): 421–432, 10.1093/intqhc/mzn043. 18801752

[2] T. H. Gallagher , A. D. Waterman , A. G. Ebers , V. J. Fraser , and W. Levinson , “Patients’ and Physicians’ Attitudes Regarding the Disclosure of Medical Errors,” Journal of the American Medical Association 289, no. 8 (2003): 1001–1007, 10.1001/jama.289.8.1001. 12597752

[3] C. W. Duclos , M. Eichler , L. Taylor , et al., “Patient Perspectives of Patient‐Provider Communication After Adverse Events,” International Journal for Quality in Health Care 17, no. 6 (2005): 479–486, 10.1093/intqhc/mzi065. 16037100

[4] R. Lamb , “Open Disclosure: The Only Approach to Medical Error,” Quality and Safety in Health Care 13, no. 1 (2004): 3–5, 10.1136/qshc.2003.008631. 14757787 PMC1758054

[5] D. Rathnayake , A. Sasame , A. Radomska , É. Ni Shé , E. McAuliffe , and A. De Brún , “What Can We Learn From Patient and Family Experiences of Open Disclosure and How They Have Been Evaluated? A Systematic Review,” BMC Health Services Research 25, no. 1 (2025): 238, 10.1186/s12913-025-12388-3. 39939873 PMC11817258

[6] K. M. Mazor , S. R. Simon , and J. H. Gurwitz , “Communicating With Patients About Medical Errors: A Review of the Literature,” Archives of Internal Medicine 164, no. 15 (2004): 1690, 10.1001/archinte.164.15.1690. 15302641

[7] G. B. Hickson , R. C. Boothman , A. M. Krumm , and R. Wyatt , “Communication and Resolution Programs Expose Hard‐to‐Hear Truths,” Frontiers in Health Services 4 (2024): 1523363, 10.3389/frhs.2024.1523363. 40103679 PMC11915142

[8] National Reporting and Learning Service , Being Open: Communicating Patient Safety Incidents With Patients and Their Carers, 2009.

[9] Australian Commission on Safety and Quality in Health Care (ACSQHC) , Australian Open Disclosure Framework: Better Communication, a Better Way to Care (Sydney: ACSQHC, 2013).

[10] J. Bryant , M. Carey , R. Sanson‐Fisher , H. Turon , A. Wei , and B. Kuss , “The Patients’ Perspective: Hematological Cancer Patients’ Experiences of Adverse Events as Part of Care,” Journal of Patient Safety 17, no. 5 (2021): 387–392, 10.1097/PTS.0000000000000347. 28306611

[11] L. Claringbold , M. Brennan , H. Lund , S. El‐Zaemey , N. Houssami , and E. Wylie , “Reflections From Women With an Interval Breast Cancer Diagnosis: A Qualitative Analysis of Open Disclosure in the BreastScreen Western Australia Program,” Asian Pacific Journal of Cancer Prevention 24, no. 2 (2023): 633–639, 10.31557/APJCP.2023.24.2.633. 36853314 PMC10162615

[12] R. Iedema , S. Allen , K. Britton , and T. H. Gallagher , “What Do Patients and Relatives Know About Problems and Failures in Care?,” BMJ Quality & Safety 21, no. 3 (2012): 198–205, 10.1136/bmjqs-2011-000100. 22178930

[13] E. O'Connor , H. M. Coates , I. E. Yardley , and A. W. Wu , “Disclosure of Patient Safety Incidents: A Comprehensive Review,” International Journal for Quality in Health Care 22, no. 5 (2010): 371–379, 10.1093/intqhc/mzq042. 20709703

[14] N. E. Ross and W. J. Newman , “The Role of Apology Laws in Medical Malpractice,” Journal of the American Academy of Psychiatry and the Law 49, no. 3 (2021): 406–414, 10.29158/JAAPL.200107-20. 34011538

[15] J. K. O'Hara , C. Reynolds , S. Moore , et al., “What Can Patients Tell Us About the Quality and Safety of Hospital Care? Findings From a UK Multicentre Survey Study,” BMJ Quality & Safety 27, no. 9 (2018): 673–682, 10.1136/bmjqs-2017-006974. PMC6109253 29545325

[16] Y. Birks , R. Harrison , K. Bosanquet , et al., “An Exploration of the Implementation of Open Disclosure of Adverse Events in the UK: A Scoping Review and Qualitative Exploration,” Health Services and Delivery Research 2, no. 20 (2014): 1–196, 10.3310/hsdr02200. 25642494

[17] Safer Care Victoria . Victorian Duty of Candour Guidelines. 2022.

[18] R. Harrison , C. Adams , N. B. Haque , et al., “Application of the Statutory Duty of Candour in the Management of Patient Safety Events: Systematic Review and Narrative Synthesis,” Journal of Patient Safety 22, no. 1 (2026): 67–72, 10.1097/PTS.0000000000001420. 40948331

[19] O. Quick , “Duties of Candour in Healthcare: The Truth, the Whole Truth, and Nothing but the Truth?” Medical Law Review 30, no. 2 (2022): 324–347, 10.1093/medlaw/fwac004. 35312762 PMC9155592

[20] Parliament of Victoria . Health Legislation Amendment (Quality and Safety) Act 2022. Parliament of Victoria; 2022.

[21] R. J. Lewicki , B. Polin , and R. B. Lount , “An Exploration of the Structure of Effective Apologies,” Negotiation and Conflict Management Research 9, no. 2 (2016): 177–196, 10.1111/ncmr.12073.

[22] M. J. Ottosen , E. W. Sedlock , A. O. Aigbe , S. K. Bell , T. H. Gallagher , and E. J. Thomas , “Long‐Term Impacts Faced by Patients and Families After Harmful Healthcare Events,” Journal of Patient Safety 17, no. 8 (2021): e1145–e1151, 10.1097/PTS.0000000000000451. 29346175 PMC6050155

[23] Department of Health & Social Care . Duty of Candour Review. Gov.uk. November 26, 2024, accessed July 16, 2026, https://www.gov.uk/government/calls‐for‐evidence/duty‐of‐candour‐review/duty‐of‐candour‐review.

[24] R. Harrison , C. Adams , N. B. Haque , et al., “A Mixed Methods Evaluation of the Statutory Duty of Candour in Victorian Health Services: Study Protocol,” Health Expectations 28, no. 1 (2025): e70180, 10.1111/hex.70180. 39936574 PMC11815560

[25] Australian Commission on Safety and Quality in Health Care . Patient Centred Care: Improving Quality and Safety through Partnerships With Patients and Consumers. 2011, accessed February 15, 2025, www.safetyandquality.gov.au.

[26] M. Walton , R. Harrison , J. Smith‐Merry , et al., “Disclosure of Adverse Events: A Data Linkage Study Reporting Patient Experiences Among Australian Adults Aged ≥45 Years,” Australian Health Review 43, no. 3 (2019): 268–275, 10.1071/AH17179. 29695314

[27] V. Braun and V. Clarke , “Reflecting on Reflexive Thematic Analysis,” Qualitative Research in Sport, Exercise and Health 11, no. 4 (2019): 589–597, 10.1080/2159676X.2019.1628806.

[28] Te Tāhū Hauora Health Quality & Safety Commission . Healing, Learning and Improving From Harm: National Adverse Events Policy (Te Tāhū Hauora Health Quality & Safety Commission, 2023).

[29] G. Tomaselli , S. C. Buttigieg , A. Rosano , M. Cassar , and G. Grima , “Person‐Centered Care From a Relational Ethics Perspective for the Delivery of High Quality and Safe Healthcare: A Scoping Review,” Frontiers in Public Health 8 (2020): 44, 10.3389/fpubh.2020.00044. 32211362 PMC7067745

[30] J. Kok , D. de Kam , I. Leistikow , K. Grit , and R. Bal , “Epistemic Injustice in Incident Investigations: A Qualitative Study,” Health Care Analysis 30, no. 3/4 (2022): 254–274, 10.1007/s10728-022-00447-3. 35639265 PMC9741561

[31] N. Gillespie and G. Dietz , “Trust Repair After an Organization‐Level Failure,” Academy of Management Review 34, no. 1 (2009): 127–145, 10.5465/AMR.2009.35713319.

[32] K. Rudolph , “Ethical Considerations in Trauma‐Informed Care,” Psychiatric Clinics of North America 44, no. 4 (2021): 521–535, 10.1016/j.psc.2021.07.001. 34763786

[33] E. Goldstein , B. Chokshi , G. Melendez‐Torres , A. Rios , M. Jelley , and A. Lewis‐O'Connor , “Effectiveness of Trauma‐Informed Care Implementation in Health Care Settings: Systematic Review of Reviews and Realist Synthesis,” Permanente Journal 28, no. 1 (2024): 135–150, 10.7812/TPP/23.127. 38444328 PMC10940237

[34] D. L. Aubin , A. Soprovich , F. Diaz Carvallo , D. Prowse , and D. Eurich , “Support for Healthcare Workers and Patients After Medical Error Through Mutual Healing: Another Step Towards Patient Safety,” BMJ Open Quality 11, no. 4 (2022): e002004, 10.1136/bmjoq-2022-002004. PMC9730392 36588324

[35] NSW Clinical Excellence Commission, Dedicated Family Contact (fact sheet), August 2020, accessed 18 March 2026, https://www.cec.health.nsw.gov.au/__data/assets/pdf_file/0009/615654/Dedicated‐family‐contact‐Fact‐sheet‐for‐staff.pdf.

[36] R. Harrison , Y. Birks , K. Bosanquet , and R. Iedema , “Enacting Open Disclosure in the UK National Health Service: A Qualitative Exploration,” Journal of Evaluation in Clinical Practice 23, no. 4 (2017): 713–718, 10.1111/jep.12702. 28220984

[37] R. Harrison , M. Walton , J. Smith‐Merry , E. Manias , and R. Iedema , “Open Disclosure of Adverse Events: Exploring the Implications of Service and Policy Structures on Practice,” Risk Management and Healthcare Policy 12 (2019): 5–12, 10.2147/RMHP.S180359. 30774487 PMC6350650

