# Supplementary material for: Patient and Family Perspectives on Statutory Duty of Candour Following Serious Adverse Patient Safety Events
Source: Health Expect. 2026 Aug 31;29(5):e70800. doi: 10.1111/hex.70800 (PMC13527863; doi:10.1111/hex.70800)
Supplement: Supplementary file 1 [file HEX-29-e70800-s001.docx]

**Appendix**

**Patient Survey — Healthcare Incident Management**

Statutory Duty of Candour Evaluation

**Survey Information**

Thank you for participating in this survey about incidents in healthcare settings. In this survey, you will be asked about your views and experiences of how healthcare staff inform patients and their families when something goes wrong during their care.

In this survey, you will:

• Answer questions by choosing from a list of options.

• Add any extra comments that you think are important.

The survey will take about 15–20 minutes to complete. Your participation is voluntary and anonymous. By answering these questions, you consent that your answers can be used to help understand the current situation for healthcare workers, patients, and their families about the Statutory Duty of Candour (SDC), how it affects health systems, and any challenges in reporting serious adverse patient safety events in Victorian hospitals.

If you have any questions, please contact Corey Adams: corey.adams@mq.edu.au

This survey will be open until: ________________________

**Section 1: Healthcare Incidents**

**Definition:** A 'healthcare incident' is "an event or circumstance during health care caused by the hospital which could have or did result in unintended or unnecessary harm to you." If you have had more than one healthcare incident, please answer these questions about the one that affected you the most.

**1.** Have you ever experienced a healthcare incident?

| ☐ | Yes |
| --- | --- |
| ☐ | No |

**2.** Where did the healthcare incident occur?

| ☐ | Public hospital |
| --- | --- |
| ☐ | Private hospital |
| ☐ | Other |

**3.** Briefly describe the healthcare incident, including what happened to you.

*Please write your answer here:*

**4.** How serious were the effects of the incident?

| ☐ | No effects |
| --- | --- |
| ☐ | Mild effects |
| ☐ | Moderate effects |
| ☐ | Severe effects |

**5.** When did you first know about the healthcare incident?

| ☐ | Immediately after it happened |
| --- | --- |
| ☐ | Within 24 hours after |
| ☐ | Within 2 days (24 to 48 hours) after |
| ☐ | 3 to 7 days after |
| ☐ | More than 7 days after |
| ☐ | Not sure |

**6.** Who first noticed the healthcare incident?

| ☐ | You |
| --- | --- |
| ☐ | Support person (e.g. family, carer) |
| ☐ | Hospital staff |

**7.** If possible, briefly explain what you remember being told.

*Please write your answer here:*

**8.** Did you feel that the doctors, nurses, and other hospital staff were open and honest with you about the healthcare incident?

| ☐ | Yes |
| --- | --- |
| ☐ | No |
| ☐ | Not sure |

**9.** Please explain your answer.

*Please write your answer here:*

**10.** How many incident management meetings did you have? (Please write the number of meetings)

*Number of meetings: _______________*

**11.** Were you satisfied with the number of incident management meetings you had?

| ☐ | Yes |
| --- | --- |
| ☐ | No |

**12.** Is there anything you would like to change about the way the healthcare incident was managed?

*Please write your answer here:*

**13.** Are there any other comments you would like to make about how the incident was managed?

*Please write your answer here:*

**14.** Would you like to take part in a short interview about your healthcare incident? If so, please provide your name and phone number and we will contact you.

*Name: ___________________________ Phone: __________________________*

**Section 2: Statutory Duty of Candour**

**What is the Statutory Duty of Candour (SDC)?** SDC is a law in Victoria that all healthcare providers must follow. SDC means that if something serious goes wrong with your care, the healthcare provider must tell you and your family about it. They must also apologise, explain what happened, and tell you how they will stop it from happening again.

**15.** Before taking this survey, did you know about the Statutory Duty of Candour (SDC)?

| ☐ | Yes, I knew about SDC. |
| --- | --- |
| ☐ | I have heard of it, but I am not sure what it is. |
| ☐ | No, I did not know about SDC. |

**16.** Do you think that the Statutory Duty of Candour is useful for acknowledging healthcare incidents to patients and their families?

| ☐ | Yes |
| --- | --- |
| ☐ | No |
| ☐ | Unsure |

**17.** Please rate the following statements about the Statutory Duty of Candour (SDC):

| **Statement** | **Strongly Disagree** | **Disagree** | **Neither Agree nor Disagree** | **Agree** |
| --- | --- | --- | --- | --- |
| SDC is useful for patients and family to find out what happened. | ☐ | ☐ | ☐ | ☐ |
| SDC is useful for improving transparency about healthcare incidents. | ☐ | ☐ | ☐ | ☐ |
| SDC is useful for improving accountability of healthcare organisations for healthcare incidents. | ☐ | ☐ | ☐ | ☐ |
| SDC is a useful approach for the healthcare professional responsible to apologise. | ☐ | ☐ | ☐ | ☐ |
| SDC is useful for building trust between clinicians and patients. | ☐ | ☐ | ☐ | ☐ |
| SDC is useful for patients to know about how the healthcare organisation will improve their services. | ☐ | ☐ | ☐ | ☐ |

**18.** Please provide further comments to explain your responses.

*Please write your answer here:*

**19.** How do you think the Statutory Duty of Candour process could be improved for patients and their families?

*Please write your answer here:*

**Section 3: Your Information**

**20.** Your age:

| ☐ | 20 years or below |
| --- | --- |
| ☐ | 21 to 30 years |
| ☐ | 31 to 64 years |
| ☐ | 65 to 84 years |
| ☐ | 85 years or older |

**21.** How do you describe your gender?

| ☐ | Man or male |
| --- | --- |
| ☐ | Woman or female |
| ☐ | Non-binary |
| ☐ | Prefer not to answer |
| ☐ | Other |

**22.** Which of the following best describes you?

| ☐ | Patient |
| --- | --- |
| ☐ | Carer |
| ☐ | Member of the public |
| ☐ | Consumer representative |
| ☐ | Other |

**Thank You**

Thank you for completing this survey!

We understand that it may be distressing to talk about hospital incidents. If you are experiencing any stress or anxiety after completing this survey, please contact:

• Lifeline: 13 11 14

• Beyond Blue: 1300 224 643

If you have any further questions, please contact Corey Adams, Clinical Researcher (Macquarie University): corey.adams@mq.edu.au

**Semi-structured Interview questions**

**1. Have you ever experienced any healthcare incident/s?**

If yes, can you tell a bit about the incident please?

If not, please go to question 3.

**2. If a patient has been harmed by mistake during treatment, what should be done by the hospital?** *(Examples: medication errors, error during surgical procedure, wrong diagnosis).*

**3. Have you ever heard of statutory duty of candour (SDC)?**

If yes, then briefly describe.

**4. What are your views about having a legal duty of candour in a healthcare organization?**

- *Prompt: What do you think this will mean for patients and families in Victoria?*
- *Prompt: What are the actual or anticipated/perceived impacts of SDC?*
- *Prompt: To what extent does SDC increase organisational accountability?*
- *Prompt: Is SDC making a difference to the management of healthcare safety events for patients and families?*

**5. What are your perceptions of the barriers for SDC to have an impact in healthcare?**

- *Prompt: What makes it difficult for healthcare providers to apologise if there is an incident?*
